# Mass spectrometry data from identification of host-defense related proteins using label-free quantitative proteomic analysis of milk whey from cows with *Staphylococcus aureus* subclinical mastitis

**DOI:** 10.1016/j.dib.2019.01.013

**Published:** 2019-01-10

**Authors:** Shaimaa Abdelmegid, Jayaseelan Murugaiyan, Mohamed Abo-Ismail, Jeff Caswell, David Kelton, Gordon Kirby

**Affiliations:** a Department of Biomedical Sciences, Ontario Veterinary College, University of Guelph, Guelph, Canada; b Freie Universität Berlin, Institute of Animal Hygiene and Environmental Health, Centre for Infectious Medicine, Berlin, Germany; c Department of Animal Biosciences, Ontario Agriculture College, University of Guelph, Guelph, Canada; d Department of Pathobiology, Ontario Veterinary College, University of Guelph, Guelph, Canada; e Department of Population Medicine, Ontario Veterinary College, University of Guelph, Guelph, Canada

## Abstract

This dataset is associated with our research article ‘Identification of host-defence related proteins using label-free quantitative proteomic analysis of milk whey from cows with *Staphylococcus aureus* Subclinical mastitis’ published in International Journal of Molecular Sciences. Milk samples were collected from cows suffering from *S. aureus*-associated subclinical mastitis and the proteins abundance were identified in comparison with samples collected from the control cows using liquid chromatography-mass spectrometry (LC-MS)-based label free proteomics analysis. Following the MS measurements, the raw spectra were processed using MaxQuant-Andromeda software and the protein identification was carried out through a search against Uniprot FASTA files of the *Bos taurus* reference proteome. Perseus software analysis was applied for computation of protein abundance. The raw file presented in this DiB was deposition at PRIDE repository in the PRIDE repository with the dataset identifier PXD007516.

## Specifications table

**Table:** 

Subject area	*Biology*
More specific subject area	*Label-free quantitative proteomics*, *Staphylococcus aureus*, *Bovine mastitis, cow milk, whey samples*
Type of data	*Raw data, table and Excel output files*
How data was acquired	*Mass spectrometry data acquired using Orbitrap Elite™ Hybrid Ion Trap-Orbitrap Mass Spectrometer coupled with an EASY-nLC™ 1200 system (ThermoFisher Scientific).*
Data format	*Raw and excel sheet*
Experimental factors	*Milk samples were collected from normal and cows suffering from sub-clinical associated with Staphylococcus aureus. The whey samples were separated, whey proteins subjected to trypsin digestion and label free quantitative analysis using liquid chromatography-mass spectrometry (LCMS).*
Experimental features	*Milk sample collection, assessment of sub-clinical mastitis status, whey separation, protein estimation, trypsin digestion and mass spectrometry analysis using LCMS method. MaxQuant software was used for protein identification through search against FASTA file of the Bos taurus reference proteome and identification of comparative protein abundance out using Perseus software.*
Data source location	*University of Guelph, Guelph, Ontario, Canada*
Data accessibility	*Data available at PRIDE:*PXD007516
Related research article	S. Abdelmegid, J. Murugaiyan, M. Abo-Ismail, J. L. Caswell, D. Kelton, G. M. Kirby. Identification of host defense-related proteins using label-free quantitative proteomic analysis of milk whey from cows with *Staphylococcus aureus* subclinical mastitis. Int J Mol Sci. (2017), 19(1): 78

## Value of the data

• Comparative listings of proteins associated with *S. aureus* associated subclinical mastitis are useful to those examining the relative abundance of host defense proteins in milk during intramammary infection
• Availability of raw LC-MS data of whey samples isolated from normal and *S. aureus* associated sub-clinical mastitis milk samples to other researchers
• This raw data is useful in direct comparison with milk samples collected with similar or different diseases.

## 1 Data

**Fig. 1:**
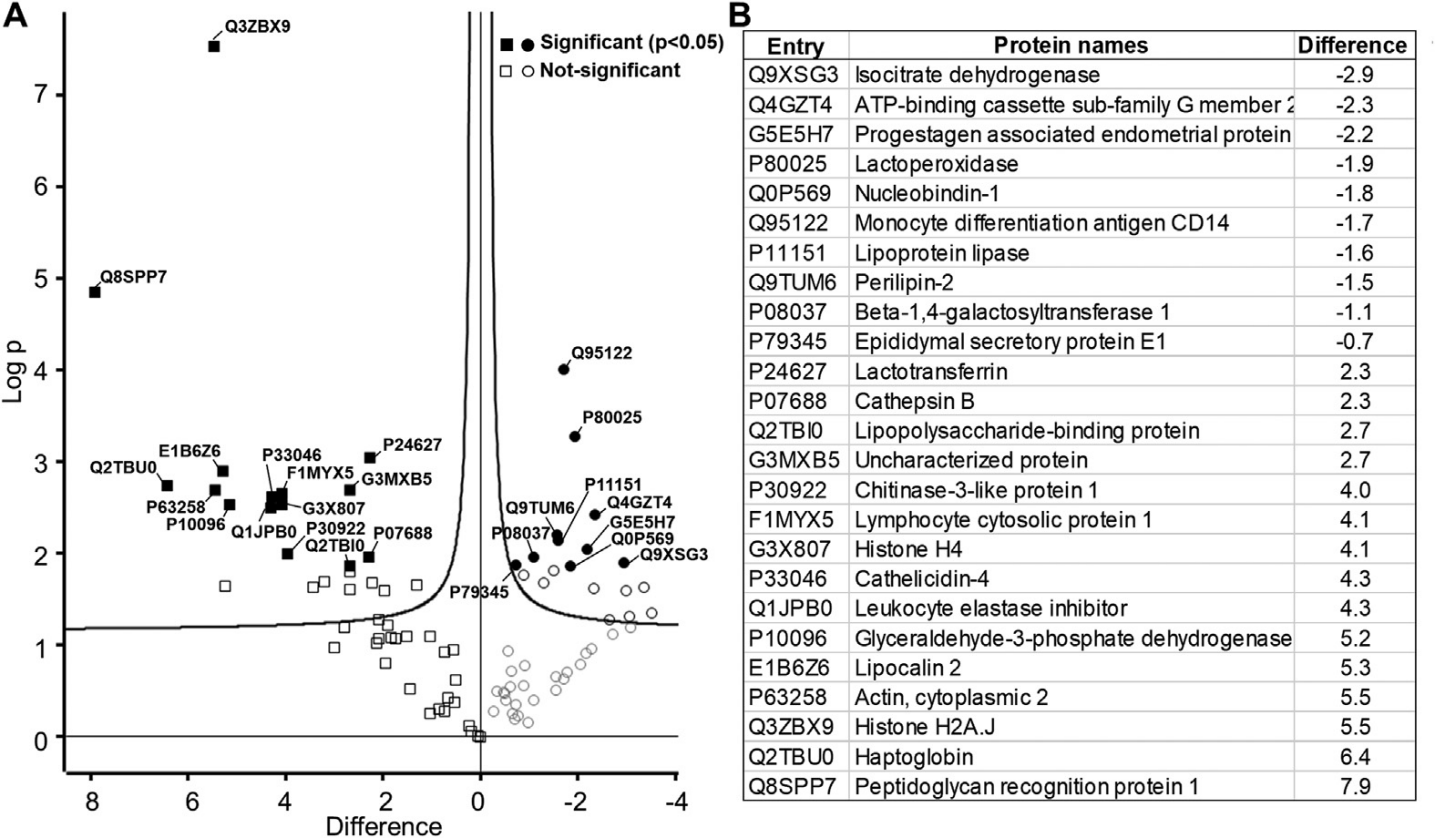
**(A)** Volcano plot comparing the milk whey control and *S. aureus* associated *sub-clinical mastitis sample groups.* The *p*-values were calculated by Student t-test analysis using log_2_ transformed peptide intensities. (**B**) List of the proteins identified as significantly differentially abundant.

**Fig. 2:**
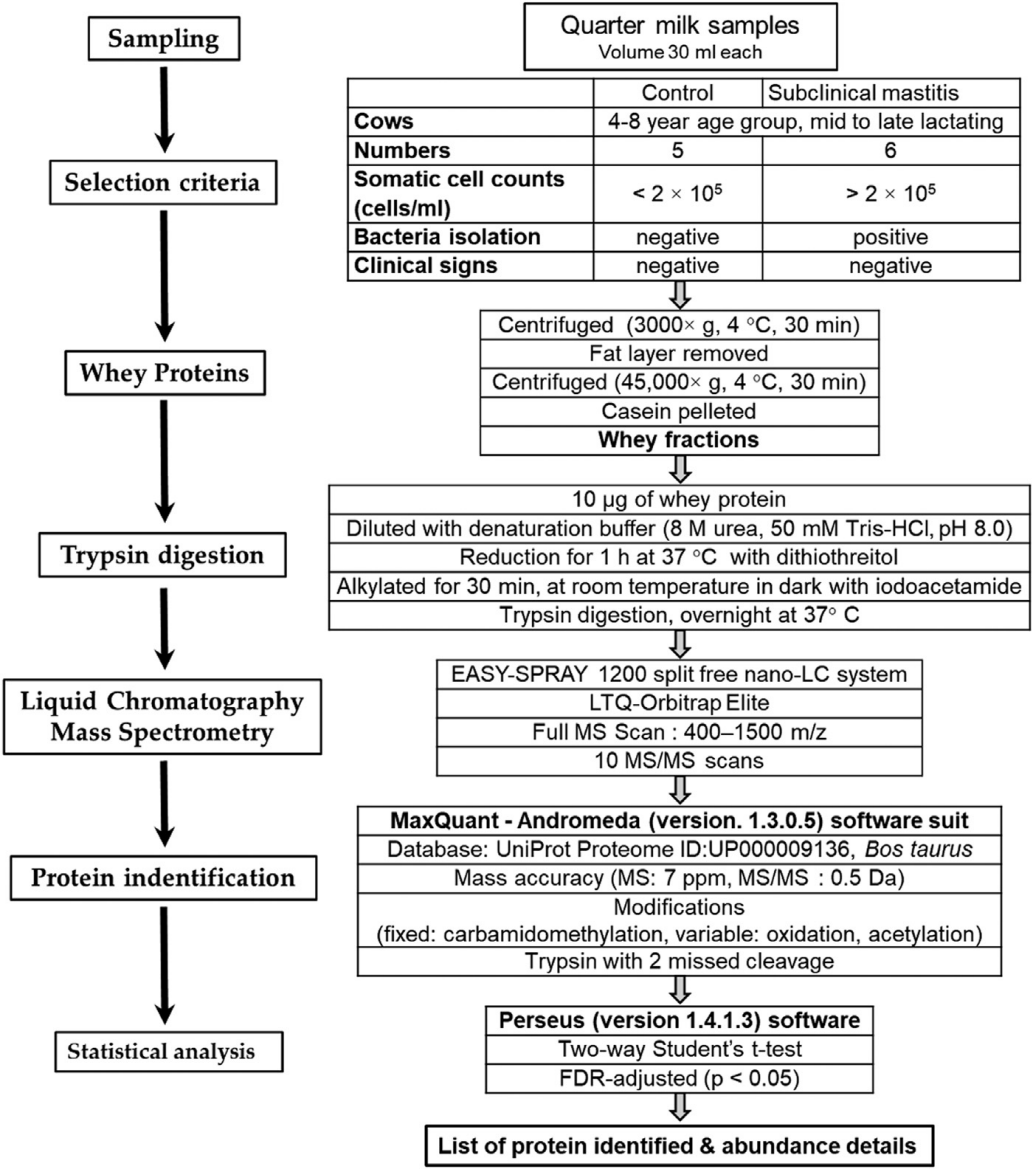
Experimental design and work flow.

This mass spectrometry data-in-brief is associated with the research article aimed at identification of protein abundantly present in milk samples of cows suffering from sub-clinical mastitis due to *S. aureus* [1]. The raw MS data and associated files can freely downloadable from the PRIDE repository with an identified PXD007516 [2]. The identified proteins are listed in a Supplementary Table, while those proteins identified as differentially abundant proteins discussed in Abdelmegid et al. [1] are shown in Fig. 1. Log^2^-fold changes range from a 2.9-fold reduction (Q9XSG3: Isocitrate dehydrogenase) to a 7.9-fold increase (Q8SPP7: Peptidoglycan recognition protein 1). Fig. 2 illustrates the steps involved in preparation of the milk samples and analysis, identification and quantification of the whey proteins.

## 2 Experimental design, materials and methods

Fig. 2 depicts the experimental design and work flow of the data discussed in Abdelmegid et al. [1]. In brief, milk was collected from dairy cows that were classified into two groups: healthy cows to serve as controls and cows with subclinical mastitis associated with *S. aureus*. Label-free quantitative proteomics analysis was applied for identification of proteins and their comparative abundance among the control and mastitis groups.

## 3 Milk samples and classification

Milk samples were collected from dairy cows at the Elora Research Station- Diary Facility at the University of Guelph in accordance to the guidelines set by the Animal Care and Use Committee of the University of Guelph. (AUP # 1424, Approved time line – Monday, April 30, 2012 to Monday, July 11, 2016). The cows were classified as being either healthy or having subclinical mastitis based on history, physical examination and testing of the milk samples by Somatic Cell Counting and bacterial culture.

## 4 Mass spectrometry analysis

Whey protein was prepared by removing fat and casein micelles by centrifugation followed by trypsin digestion. Separation of peptides was carried out by liquid chromatography and an Orbitrap-based mass spectrometry system (Orbitrap Elite™ Hybrid Ion Trap coupled with an EASY-nLC™ 1200 system; ThermoFischer Scientific) as described in [1].

## 5 Data analysis

MaxQuant-Andromeda software suit was utilised to process the raw spectra and to search against the *Bos taurus* protein reference proteomes (UniProt Proteome ID: UP000009136). Label-free quantification of identified proteins was performed and statistically significant differences in protein abundance in the subclinical group in comparison to that of the control group was determined using Perseus 1.4.1.3 software (http://141.61.102.17/perseus_doku/doku.php?id=start) [1], [3], [4], [5].

## 6 Mass spectrometry dataset deposit

The mass spectrometry data was deposited at the ProteomeXchange (PX) Consortium [2] via the PRIDE (PRoteomics IDEntifications) partner repository and is accessible with the dataset identifier PXD007516.
